# *In Vitro* Antimycobacterial Activity Evaluation of a New Lead Compound (LQFM326) against Clinical Strains of *Mycobacterium* sp.

**DOI:** 10.1021/acsomega.5c04174

**Published:** 2025-08-26

**Authors:** Tracy M. M. Martins, Luciano M. Lião, Gerlon A. R. Oliveira, Pedro E. A Silva, Ana J. Reis, Yasmin C. Neves, Glaura R. C. C. Lima, Beatriz S. Gontijo, José R. do Carmo Neto, Jonathas X. Pereira, André Kipnis, Ricardo Menegatti

**Affiliations:** † Federal University of Pará, Faculty of Medicine, Center for Morphological Studies (NEM-ATM), 68372-040 Altamira, PA, Brazil; ‡ Federal University of Goiás, Institute of Chemistry, Nuclear Magnetic Resonance Laboratory (LabRMN), 74690-900 Goiânia, GO, Brazil; § University of Brasília, Department of Pharmacy, Faculty of Health Sciences, 70910-900 Brasília, DF, Brazil; ∥ Federal University of Rio Grande, Faculty of Medicine, Laboratory of Mycobacteria, 96203-900 Rio Grande, RS, Brazil; ⊥ Medical Biology of Central Public Health Laboratory of the Federal District, 70830-010 Brasília, DF, Brazil; # Federal University of Goiás, Institute of Tropical Pathology and Public Health, Cellular and Molecular Pathology Laboratory, 74690-900 Goiânia, GO, Brazil; ∇ Federal University of Goiás, Institute of Tropical Pathology and Public Health, Molecular Bacteriology Laboratory, 74690-900 Goiânia, GO, Brazil; ○ Federal University of Goiás, Faculty of Pharmacy, Laboratory of Medicinal Pharmaceutical Chemistry (LQFM), 74001-970 Goiânia, GO, Brazil

## Abstract

Tuberculosis (TB) remains a significant global public health challenge. The novel compound LQFM326 was evaluated for its antimycobacterial activity against seven Mycobacterium species. Minimum inhibitory concentrations (MICs) were determined, revealing values of 15.6 μg/mL against *Mycobacterium tuberculosis* H37Ra and 12.5 μg/mL against clinical strains. The MIC values observed for these reference antimicrobials against *M. tuberculosis* H37Ra were 0.25 μg/mL for rifampicin and 0.125 μg/mL for isoniazid. Surface damage to *Mycobacterium abscessus* cells was observed *via* scanning electron microscopy (SEM), confirming morphological alterations induced by LQFM326. Cellular viability was assessed using the Live/Dead assay, with a CC_50_ of 126.68 ± 42.66 μg/mL. The selectivity index (SI), calculated from MIC and CC_50_ values, ranged from 2.03 to 10.13, with values above 10 indicating favorable selectivity. Additionally, synergistic effects were observed when LQFM326 was combined with other antibiotics. These findings highlight LQFM326 as a promising antimycobacterial agent with potential efflux-inhibitory and synergistic properties. Further studies are needed to validate its efficacy across diverse clinical strains and to elucidate its mechanism of action.

## Graphical abstract

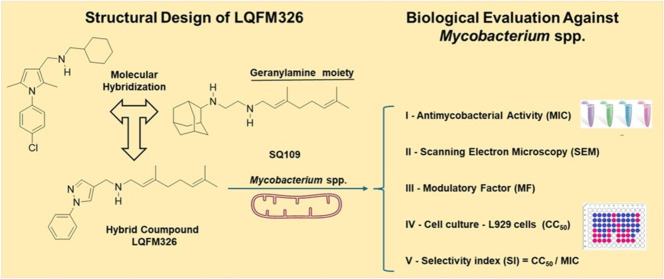

## 1 Introduction

Bacteria of the *Mycobacterium* genus possess a cell wall enriched with mycolic acids, which constitute up to 60% of their dry weight, contributing to their low permeability. These mycobacteria are classified into slow-growing species, such as *Mycobacterium tuberculosis*, the primary causative agent of tuberculosis (TB), and fast-growing species, such as the *Mycobacterium abscessus* complex (MABC) and *Mycobacterium smegmatis*.^1^

The global prevalence of nontuberculous mycobacteria (NTM) infections has been increasing. These infections are also associated with high rates of resistance to multiple classes of antibiotics.^2–4^ Primary infections caused by NTM include skin and subcutaneous tissue infections, lung infections, postsurgical infections, and systemic infections in immunocompromised individuals, particularly those with chronic lung diseases or cystic fibrosis. Among NTM species, the most commonly isolated in humans worldwide are members of the *Mycobacterium avium* complex (MAC) and MABC.^5,6^

*M. tuberculosis* belongs to the *M. tuberculosis* complex (MTBC) and is the primary species responsible for causing tuberculosis (TB) in humans, with an estimated 10 million new cases and 1.4 million deaths each year, tuberculosis continues to represent one of the leading causes of morbidity and mortality globally.

Additionally, drug-resistant TB has become a significant public health issue. In 2023, it was reported that 400,000 people worldwide developed rifampicin-resistant TB (RR-TB) or multidrug-resistant TB (MDR), which is resistant at least to both rifampicin and isoniazid. The 2024 WHO Bacterial Priority Pathogens List (WHO BPPL) identifies 24 antibiotic-resistant pathogens across 15 bacterial families, with *M. tuberculosis* being a central focus. This list serves as a strategic framework to prioritize research and development (R&D) efforts and to guide investments aimed at addressing antimicrobial resistance (AMR).^2–4,7^ The primary infections caused by NTM include skin and subcutaneous tissue infections, lung infections, postsurgical infections, and systemic infections in immunocompromised patients, particularly those with chronic lung diseases or cystic fibrosis. Among NTM species, the most frequently isolated in humans worldwide are members of the MAC and MAB.^5,6^

Given the rising prevalence and high rates of antimicrobial resistance in mycobacterial infections, the R&D of new lead compounds for use as antimycobacterial agents or as adjuvants in treating these infections represents a significant challenge. Membrane proteins known as Mycobacterial membrane protein Large (MmpL) constitute a family of proteins that play essential roles in the transport of lipids, polymers, and immunomodulatory molecules.^8^ They are also involved in the efflux of therapeutic drugs, making them among the most promising targets for new tuberculosis treatments identified in recent years. Several small-molecule inhibitors of MmpL3 - such as SQ109 (**1**),^9,10^ phenylpyrrole (**2**),^11^ BM635 (**3**),^12^ phenylpyrazole (**4**),^12^ and ZINC248146645 (**5**)^13^ -have been identified as novel lead compounds targeting this protein (Figure 1). Although no approved anti-TB drugs currently act on this target, MmpL3 is considered a highly promising candidate for future drug development.

**Figure 1:**
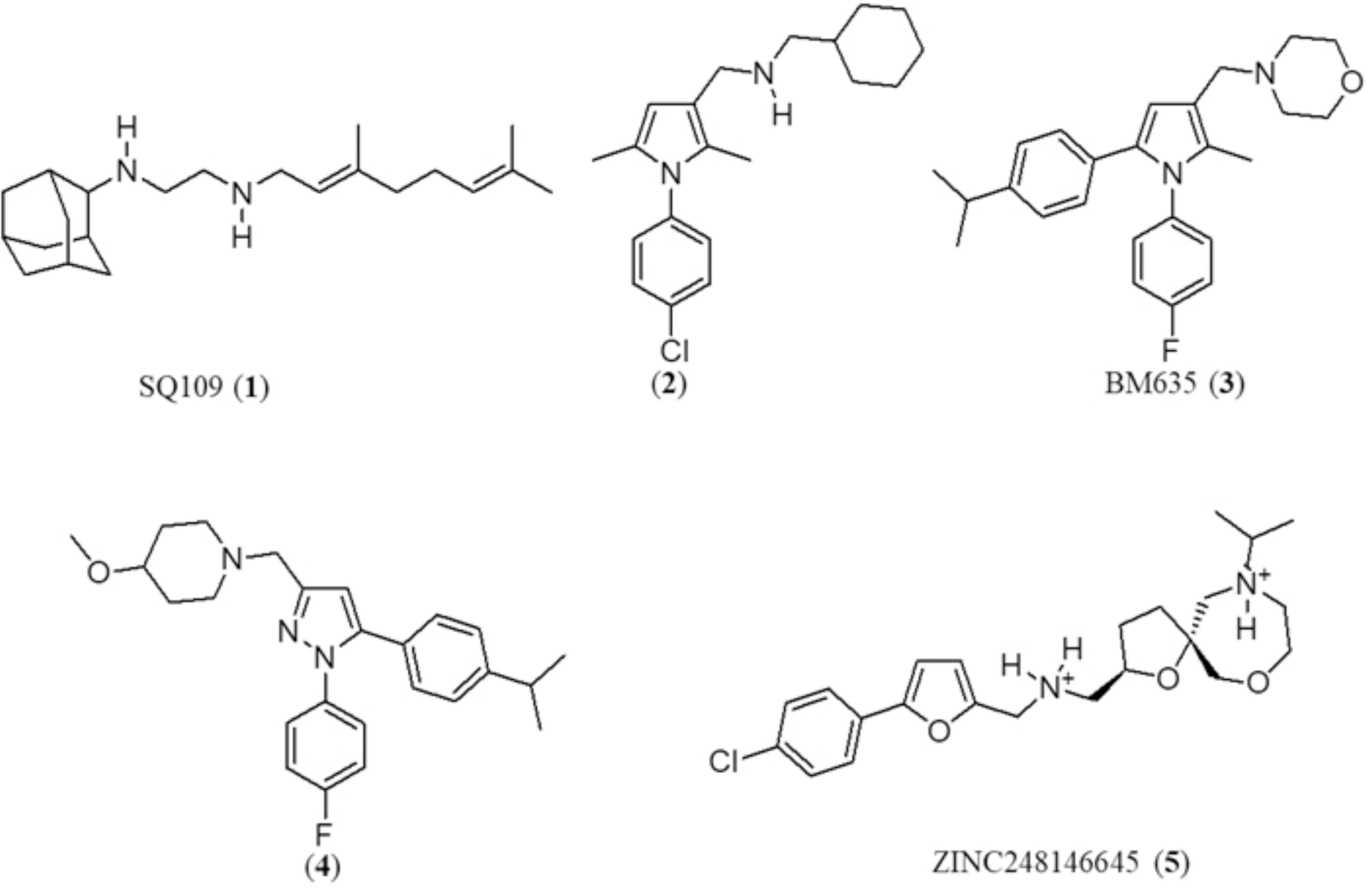
Small-molecule inhibitors of MmpL3.

This study reports the structural design and synthesis of a novel heterocyclic compound, LQFM326 (**3**), derived from the lead structures (**1**)^9^ and SQ109 (**2**),^11^ employing the molecular hybridization strategy. As depicted in Figure 2A, A and B the structural moiety present in compound (**2**) was replaced by its bioisostere, 1-(phenyl)-1*H*-pyrazol-4-yl (A and B), in the design of LQFM326 (**6**), while the geranylamine (**8**) moiety from compound (**1**) was retained. The LQFM326 (**6**) was evaluated against some *Mycobacterium* species, employing minimum inhibitory concentration (MIC) and modulatory factor (MF) assays. To further investigate the damage to the surface of *M. abscessus* in the presence of LQFM326 (**6**), scanning electron microscopy (SEM) was utilized. Additionally, cellular viability was assessed using the “Live and Dead” assay in the murine fibroblast cell line L929, in the presence of LQFM326 (**6**). The Selectivity Index (SI) also was calculated.

**Figure 2:**
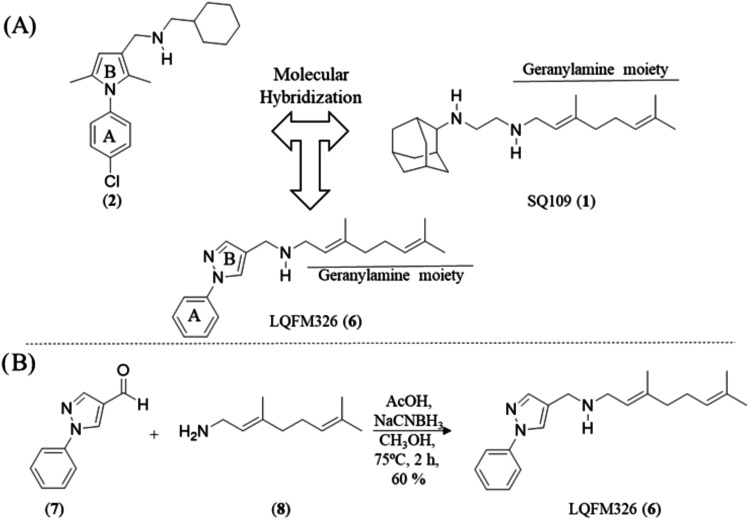
(A) Structural design of LQFM326 (**6**) from SQ109 (**1**) and phenylpyrrole (**2**). (B) Synthetic route for the preparation of (*E*)-3,7-dimethyl-*N*-((1-phenyl-1*H*-pyrazol-4-yl)­methyl)­octa-2,6-dien-1-amine (**6**) – LQFM326.

## 2 Results

### 2.1 Synthesis of LQFM326 (**6**)

Synthesis of LQFM326 (**6**) as illustrated in Figure 2B, the synthesis of (*E*)-3,7-dimethyl-*N*-((1-phenyl-1*H*-pyrazol-4-yl)­methyl)­octa-2,6-dien-1-amine (**6**) was performed *via* reductive amination conditions, achieving a yield of 60%.^14^

### 2.2 Determination of the Minimum Inhibitory Concentration (MIC)

The MIC of LQFM326 (**6**) was determined using the broth microdilution method, using resazurin as metabolic marker, as follows: 62.5 μg/mL against *M. abscessus* subsp. *massiliense*; 12.5 μg/mL against *M. abscessus* subsp. *abscessus*, *M. abscessus* subsp. *bolletii*, and *Mycobacterium intracellulare* clinical strains; 15.6 μg/mL against the *M. tuberculosis* H37Ra ATCC 25177 strain; and 12.5 μg/mL against a *M. tuberculosis* clinical strain. For *M. avium*, the MIC was ≥100 μg/mL (Table 1). The control positive showed MIC values consistent with established literature.

**Table 1:** Minimum Inhibitory Concentration (MIC) of LQFM326 (**6**) against Different Mycobacteria Strains and Selectivity Index (SI)

strains	MIC (μg/mL)	CC_50_ (μg/mL)	SI^a^
M. tuberculosis-H37Ra *ATCC 25177*	15.6	126.68 ± 42.66	8.12
M. tuberculosis clinical strain	12.5	126.68 ± 42.66	10.13
M. avium clinical strain	≥100	126.68 ± 42.66	1.27
M. intracellulare clinical strain	12.5	126.68 ± 42.66	10.13
M. abscessus subsp. *abscessus* clinical strain	12.5	126.68 ± 42.66	10.13
M. abscessus subsp. *bolletii* clinical strain	12.5	126.68 ± 42.66	10.13
M. abscessus *subsp. massiliense* GO06	62.5	126.68 ± 42.66	2.03

a SI, selectivity index = CC_50_/MIC.

### 2.3 Evaluation of the Modulatory Factors (MF)

The evaluation of LQFM326 (**6**) as an antimicrobial against *M. abscessus* subsp. *abscessus* resulted in a significant reduction in the MIC when combined with the efflux inhibitor (EI) thioridazine (TZ) (MF = 4). However, no significant MIC reduction was observed when LQFM326 (**6**) was combined with verapamil (VP) (MF = 2) nor when it was combined with clarithromycin (CLA) or ciprofloxacin (CIP) (Table 2). For the *M. intracellulare* strain, LQFM326 (**6**), when used as an antimicrobial in combination with the classic efflux inhibitors VP or TZ, showed MFs of 8 and 16, respectively. Additionally, when evaluated for interaction with commonly used antimicrobials, LQFM326 (**6**) significantly reduced the MIC of rifampicin (RMP) (MF ≥ 16). Lastly, for *M. abscessus* subsp. *bolletii*, no significant MF was observed either when LQFM326 (**6**) was used combined with efflux inhibitors nor in combination with CIP.

**Table 2:** Results of the Modulatory Factor Assay Evaluating the Influence of Efflux Inhibitors on the Antimicrobial Compound against Strains of *M. abscessus* subsp. *abscessus*, *M. abscessus* subsp. *bolletii* or *M. intracellulare*

assess	modulating factor^a^
*M. abscessus* subsp. *abscessus*	
compound and antibiotic, in association with EI	
LQFM326+ VP	2
LQFM326+ TZ	4
compound and IEM, in association with antibiotic	
CLA + LQFM326	2
CIP + LQFM326	2
*M. abscessus* subsp. *bolletii*	
compound and antibiotic, in association with EI	
LQFM326 + TZ	2
compound and IEM, in association with antibiotic	
CIP + LQFM326	1
*M. intracellulare*	
compound and antibiotic, in association with EI	
LQFM326 + VP	8
LQFM326 + TZ	16
compound and IEM, in association with antibiotic	
CLA + LQFM326	2
EMB + LQFM326	2
RMP + LQFM326	≥64

a Modulatory factors were performed only for strains/tests with MIC of compounds <100 μg/mL; **EI**: efflux inhibitor; **VP**: Verapamil; **TZ**: Thioridazine; **CLA**: Clarithromycin; **CIP**: Ciprofloxacin; **EMB**: Ethambutol; **RIF**: Rifampicin.

### 2.4 Scanning Electron Microscopy (SEM) Analysis

Colonies exposed to LQFM326 (**6**) for 24 h exhibited noticeable changes in morphology and cell surface, as shown in Figure 3. Some bacilli displayed large depressions and pores on their surfaces, suggesting potential damage to the mycobacterial cell wall. Measurements were taken from both the control colonies and the test colonies. Bacilli from the control colony had an average length of 1.89 μm (SD ± 0.39 μm) and a thickness of 0.27 μm (SD ± 0.02 μm). In contrast, bacilli from the colony exposed to LQFM326 (**6**) had an average length of 1.51 μm (SD ± 0.34 μm) and a thickness of 0.36 μm (SD ± 0.03 μm). These observations indicate that exposure to LQFM326 (**6**) resulted in a slight reduction in length and an increase in thickness compared to the nonexposed colony.

**Figure 3:**
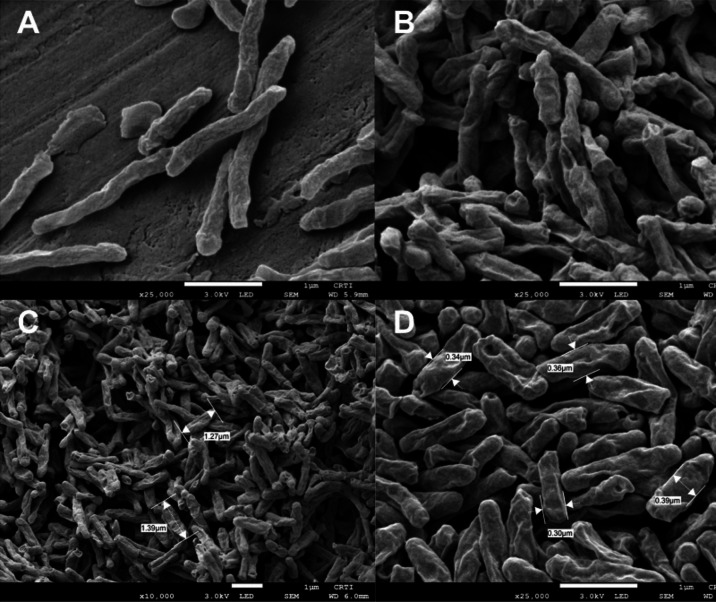
(A) Colony of *M. abscessus* subsp. *massiliense* GO06 control grown on nutrient agar. (B) Colony of *M. abscessus* subsp. *massiliense* GO06 cultured in nutrient agar and exposed to a concentration of 31.25 μg/mL of LQFM326 (**6**) for 24 h. Length (C) and thickness (D) of *M. abscessus* subsp. *massiliense* GO06 exposed to the compound LQFM326 (**6**) are shown.

### 2.5 “Live and Dead” Assay

The effect of LQFM326 (**6**) on the L929 cell line, commonly used in toxicology tests, is presented in Figure 4. Only the highest concentration assessed (41.67 μg/mL) exhibited significant toxicity, showing a statistical difference when compared to the control (0 μg/mL) (*p* = 0.0005). Furthermore, it was demonstrated that the solvent (DMSO) did not contribute to cytotoxicity, as the highest concentration resulted in higher mortality compared to the DMSO-treated group (*p* = 0.0002). The other concentrations (2.6–20.84 μg/mL) did not affect cell viability significantly, either compared to the control (*p* > 0.9587) or the DMSO-treated group (*p* > 0.8035). Consequently, the CC_50_ of LQFM326 (**6**) was determined to be 126.68 ± 42.66 μg/mL (Figure 4).

**Figure 4:**
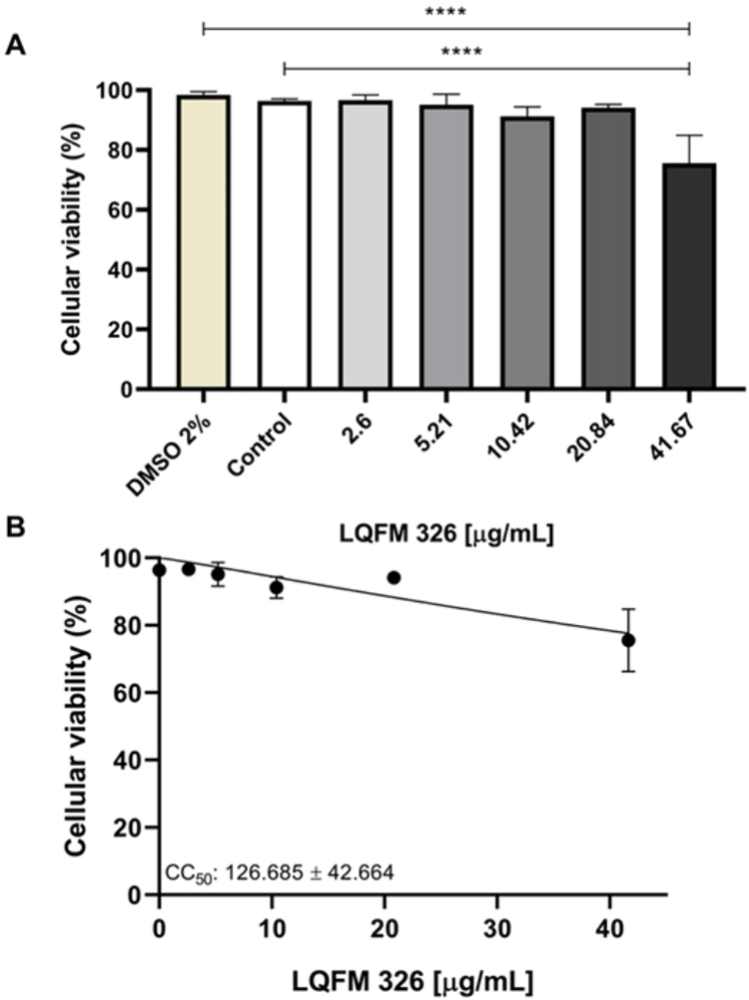
(A) CC_50_ curve. (B) Cell viability of L929 cells 24 h after exposure to LQFM326 (**6**).

### 2.6 Selectivity Index (SI)

The calculated SIs are presented in Table 1. The clinical strains of *M. tuberculosis* clinical strain, *M. intracellulare* clinical strain, *M. abscessus* subsp. *abscessus* clinical strain, and *M. abscessus* subsp. *bolletii* exhibited an SI of 10.13. The reference strain *M. tuberculosis* H37Ra (ATCC 25177) showed an SI of 8.12; *M. abscessus* subsp. *massiliense* GO06 demonstrated an SI of 2.03; and the clinical strain of *M. avium* displayed an SI of 1.27 (Table 1).

## 3 Discussion

The growing resistance to antimicrobials commonly used in the treatment of mycobacterial infections highlights the urgent need for the development of novel lead compounds with effective antimicrobial activity. Following the experimental protocols described by Costa et al. (2016),^15^ this study evaluated the involvement of efflux pumps. The compound LQFM326 (**6**) demonstrated promising antimycobacterial activity, exhibiting a minimum inhibitory concentration (MIC) of 12.5 μg/mL against clinical strains of *M. abscessus* subsp. *bolletii*, *M. abscessus* subsp. *abscessus*, *M. intracellulare*, and *M. tuberculosis* (Table 1).

The antimycobacterial activity demonstrated by LQFM326 (**6**) is particularly significant, given that TB, caused by *M. tuberculosis*, requires complex treatment regimens involving multiple drugs administered over prolonged periods. These challenging treatment protocols often result in poor patient adherence, thereby contributing to the emergence of antimicrobial resistance.^16^ Moreover, clinically relevant NTM infections—such as those assessed in this study—are frequently associated with high levels of resistance to multiple classes of antimicrobials.^4,6,17^ Consequently, there is a pressing need for the development of effective therapeutic agents to combat these infections.

Mycobacteria presents several known drug efflux mechanism knows to confer resistance for antimicrobials.^18^ Thus, we hypothesized that their presence could also interfere the cytoplasmic concentration levels of LQFM326 (**6**). Thus, mycobacteria’s efflux pump potential involvement in the observed MICs for LQFM326 (**6**) was evaluated by assaying its activity together with the efflux inhibitors VP or TZ. Interestingly, when LQFM326 (**6**) was combined with VP or TZ, a significant lowering of MICs were observed for *M. intracellulare*, resulting in modulation factors of 8 or 16, respectively. A significant decrease of MIC was also observed when TZ was assayed together with LQFM326 (**6**) against *M. abscessus* subsp. *abscessus*, MF of 4.

Although, the antimycobacterial potential of LQFM326 (**6**) showed of significance, it is also interesting to analyze if it could contribute to additional benefits by synergistically interact with known antimicrobials used in clinical practice. LQFM326 (**6**) markedly reduced the MIC of RMP against *M. intracellulare*, with an MF ≥ 16. These findings indicate that LQFM326 (**6**), beyond its potential as a direct antimycobacterial agent, may also serve as an adjuvant in mycobacterial therapy. In this context, the results suggest possible synergistic interactions with commercially available antimicrobials and future investigations may also validate if this combination could not only reduce MIC, but the regime time of treatment to avoid complications such as adverse effects and resistance development.

Although only MF ≥ 4 are considered significant,^19,20^ for the *M. abscessus* subsp. *abscessus* strain, the combination of LQFM326 (**6**) with CIP or CLA resulted in a 50% reduction in MIC. For CLA, the MIC decreased from 8 μg/mL to 4 μg/mL, altering the strain’s phenotype from resistant to intermediate resistant. In the case of CIP, the MIC dropped from 2 μg/mL to 1 μg/mL, shifting the phenotype from intermediate resistant to susceptible, according to CLSI guidelines.^21^ A 50% reduction in MIC, even when full susceptibility is not achieved, can still have a meaningful clinical impact on the treatment of mycobacterial infections. In such scenarios, adjusting antimicrobial dosages - while remaining within safe therapeutic limits—may represent a viable treatment strategy.^12,22^

Lieutaud et al.^23^ demonstrated that geranylamine (**8**) binds directly to purified AcrB, a component of the AcrAB-TolC complex, which constitutes the major tripartite efflux pump system in *Escherichia coli*. Geranylamine (**8**) exhibited efflux pump inhibitory activity *via* a noncompetitive mechanism, representing a distinct class of efflux pump inhibitors (EPIs).^23,24^ Given that geranylamine (**8**) is incorporated into the structure of LQFM326 (**6**), it is possible that LQFM326 (**6**) may also exert its effects through efflux pump inhibition, however, this mechanism requires further investigation. In addition to the geranylamine moiety (**8**), LQFM326 (**6**) also contains heteroaryl groups (A and B) (Figure 2A). As shown in Figure 1, several lead compounds—such as (**2–3**)^11,12^ and (**5**)^13^—feature heteroaryl moieties that can be considered bioisosteres. Notably, compound (**4**)^12^ possesses a phenylpyrazole moiety, which is also present in LQFM326 (**6**). Moreover, lead compounds (**2–5**) include a benzylic-like amine, which may play a key role in forming a complex with the MmpL3 target. Since LQFM326 (**6**) was developed using a molecular hybridization strategy, it may have incorporated the key structural features found in lead compounds (**1–5**). However, further studies are required to confirm this hypothesis.

The live and dead assay^25^ confirmed the effectiveness of the staining method in distinguishing viable and nonviable cells using neutral red and Evans blue dyes, as previously reported by Gomez-Gutierrez et al.^26^ and Vijayaraghavareddy et al. (2017).^27^ Using this assay, exposure of the L929 cell line to LQFM326 (**6**) yielded a CC_50_ value of 126.68 ± 42.66 μg/mL.

The relationship between CC_50_ and MIC was evaluated for all *Mycobacterium* species exposed to LQFM326 (**6**). The compound LQFM326 (**6**) exhibited greater selectivity against the clinical strains of *M. tuberculosis*, *M. intracellulare*, *M. abscessus* subsp. *abscessus*, and *M. abscessus* subsp. *bolletii*, with an SI of 10.13.^28,29^ Compounds with an SI greater than 10 are considered promising lead compounds for further investigation.^30^ Comparing these reference SI values with that of LQFM326 (**6**) would allow for a more comprehensive assessment of its safety and efficacy profile. Rifampicin, recognized as the standard first-line antituberculosis drug, exhibits a Selectivity Index (SI), indicating a favorable therapeutic window. *In vitro*, the combination of doxorubicin and rifampicin showed the highest SI of 3.43, and only rifampicin SI 2.29.^31^

Considering that LQFM326 (**6**) exhibited activity against *M. tuberculosis* (MT), showed a synergistic effect when combined with anti-MT drugs, and caused damage to the bacterial surface, these findings suggest that LQFM326 (**6**) may act as a multitarget compound. The combination of these effects may be advantageous in the search for new multitarget lead compounds for the treatment of TB.

## 4 Conclusions

LQFM326 (**6**) exhibited activity against *Mycobacterium* species, and when combined with antimicrobial drugs, a synergistic effect was observed. The SEM analysis revealed damage to *M. abscessus* subsp. *massiliense* GO06 upon exposure to LQFM326 (**6**). In the “Live and Dead” assay, LQFM326 (**6**) demonstrated a CC_50_ of 126.68 ± 42.66 μg/mL. When compared to the MIC values for four *Mycobacterium* species, the SI was calculated to be 10.13. In summary, LQFM326 (**6**) represents a promising new lead compound with an antimicrobial profile. However, further studies involving a broader range of strains, along with additional investigations into its mechanism of action, are required.

## 5 Materials and Methods

### 5.1 Chemicals

phenylhydrazine hydrochloride (Merck, MW = 144.60, >99%), 1,1,3,3-tetramethoxypropane (Aldrich, MW = 164.20, 99%), hydrochloric acid (Merck, MW = 36.46, 37%), trifluoroacetic acid (Aldrich, MW= 114.02, 98.5%), hexamethylenetetramine (Sigma-Aldrich, MW = 140.19, 99%), acetic acid (Aldrich, MW= 60.05, 99%), NaCNBH_3_ (Sigma-Aldrich, MW = 62.84, 95%), resazurin sodium salt (Aldrich, MW = 251.17, 99%), thioridazine hydrochloride (Merck, MW = 407.044, 99%), verapamil hydrochloride (Merck, MW = 326.86, 99%), clarithromycin (Merck, MW = 747.95, 99%), isoniazid (Merck, MW = 137.14, >99%), ciprofloxacin (Merck, MW = 331.34, 98%), rifampicin (Merck, MW = 822.94, 99%), ethambutol dihydrochloride (Exodo cientifica, MW = 277.23, 99%), EDTA (Merck, MW = 292.24, >98%), Fetal Bovine Serum (Merck), hexamethyldisilazane (Merck, MW = 161.39, >99%), RPMI-1640 Medium (Merck), DMSO (Merck, MW = 78.13, >99.9%), Neutral Red hydrochloride (Merck, MW = 288.78, 99%), and Evan’s Blue hydrochloride (ACS Científica, MW = 960.81, 99%).

### 5.2 Characterization and Analytical Methods

Reactions were monitored by Thin-Layer Chromatography (TLC) using commercially available precoated plates (Whatman 60 F254 silica). The developed plates were examined under UV light at wavelengths of 254 and 365 nm. Proton ^1^H and ^13^C NMR spectra were recorded in the indicated solvent on a Bruker Avance III 500 MHz spectrometer (Bruker, Germany). Chemical shifts are reported in parts per million (ppm) relative to TMS, and coupling constants are given in Hertz. All assignments of ^1^H and ^13^C NMR signals were consistent with the expected chemical structures of the products. Infrared (IR) spectra were recorded on a PerkinElmer Spectrum Bx-II FT-IR System spectrophotometer (PerkinElmer, United States), with samples prepared as films on KBr discs. Melting points were measured using a Marte melting point apparatus (Marte, Brazil), and the values were uncorrected. Organic solutions were dried over anhydrous sodium sulfate, and solvents were removed under reduced pressure using a rotary evaporator. Mass spectra (MS) were obtained with a microTOF III (Bruker Daltonics, Germany). For sample preparation, 1 μg of the sample was dissolved in 1 mL of methanol. For analysis in positive mode, 1 μL of formic acid was added to the sample. The solution was directly infused at a flow rate of 3 μL/min into the ESI source. The ESI­(+) source conditions were as follows: nebulizer with nitrogen gas at 0.4 bar, temperature at 200 °C, capillary voltage of −4 kV, transfer capillary temperature of 200 °C, drying gas flow rate of 4 L/min, end plate offset of −500 V, skimmer of 35 V, and collision voltage of −1.5 V. Each spectrum was acquired with 2 microscans. The resolving power was *m*/Δ*m*50% = 16,500.00, where Δ*m*50% is the peak full width at half-maximum. Mass spectra were acquired and processed using Data Analysis software (Bruker Daltonics, Bremen, Germany).

### 5.3 Synthesis of (*E*)-3,7-dimethyl-*N*-((1-phenyl-1*H*-pyrazol-4-yl)­methyl)­octa-2,6-dien-1-amine (**6**) −LQFM326 ^14^

To a stirred heterogeneous mixture of 1-phenyl-1*H*-pyrazole-4-carbaldehyde (**7**) (172 mg, 1.0 mmol), (*E*)-3,7-dimethylocta-2,6-dien-1-amine (**8**) (153 mg, 1.0 mmol), in 5 mL of MeOH and was adjusted to pH 5.0 by dropwise addition of concentrated acetic acid. In turn, was added NaBH_3_CN (31 mg, 0.5 mmol) in one portion and the mixture was stirred at 75 °C for 2 h. After thar, MeOH was then evaporated and the residue was partitioned between water and CH_2_Cl_2_ and the combined organic layers were dried (Na_2_SO_4_), concentrated in vacuo, and the crude product was purified by column chromatography (SiO_2_, hexane/AcOEt = 6:4) to (*E*)-3,7-dimethyl-*N*-((1-phenyl-1*H*-pyrazol-4-yl)­methyl)­octa-2,6-dien-1-amine (**6**) (185 mg, 60%) as a beige solid, m.p.= 110–112 °C, *R*_f_ = 0.71 (CH_2_Cl_2_/MeOH = 95:5). I*R*_max_ (KBr) cm^–1^: 3422 (υ N–H), 2932 (υ C–H), 2421 and 2357 (υ NH.HCl) and 1635 (υ C=C) (Figure S1, Supporting Information); ^1^H NMR (500.13 MHz) CDCl_3_/TMS (δ): 8.43 (1H, *s*, H-14), 7.75 (1H, *s*, H-11), 7.65 (2H, *m*, H-16 and 20), 7.40 (2H, *m*, H-17 and 19), 7.27 (1H, *m*, H-18), 5.40 (1H, *t*, *J* = 7.2, H-2), 5.05 (1H, *m*, H-6), 3.99 (2H, *s*, H-13), 3.50 (2H, *d*, *J* = 7.2, H-1), 2.09 (4H, *m*, H-4 and 5), 1.67 (3H, *s*, H-8), 1.61 (3H, *s*, H-9), 1.60 (3H, *s* H-10) (Figure S2 and Table 2, Supporting Information); 2D NMR (HSQC/HMBC–125.76 MHz) CDCl_3_/TMS (δ): 146.1 (C-3), 142.0 (C-11), 139.6 (C-15), 132.2 (C-7), 129.5 (C-12, 17 and 19), 129.0 (C-14), 127.0 (C-18), 123.4 (C-6), 119.2 (C-16 and 20), 113.7 (C2), 42.5 (C-1), 39.6 (C-4), 38.9 (C-13), 26.2 (C-5), 25.7 (C-8), 17.8 (C-10), 16.7­(C-9) (Figures S3 and S4 and Table 2; Supporting Information); ESI-MS calculated for C_20_H_27_N_3_ [M + H]^+^ *m*/*z* of 310.2283, found: 310.2276, Error: 0.304 (Figure S5, Supporting Information).

### 5.4 Antimicrobial Assessment: Evaluation of Antimycobacterial Activity

For the microbiological assessment, 10 mg of LQFM326 (**6**) (0.032 mmol) were dissolved in 1 mL of DMSO. The stock solution was then sequentially prepared by adding 9 mL of sterile water or broth, ensuring the final DMSO concentration did not exceed 10% (v/v), resulting in an initial concentration of 1 mg/mL. The stock solution was stored at −20 °C until use.

### 5.5 Assessment of Antimycobacterial Activity in Mycobacteria

The MIC of LQFM326 (**6**) was determined using the broth microdilution method, following standard protocols.^21^ Briefly, 2-fold serial dilutions of the LQFM326 (**6**) were prepared in 96-well plates, and bacterial strains were added to each well. The MIC was evaluated against two *M. tuberculosis* strains (H37Ra ATCC 25177 and a clinical isolate) as well as four NTM strains (*M. abscessus* subsp. *bolletii*, *M. abscessus* subsp. *abscessus*, *M. avium*, and *M. intracellulare*) from clinical sources. Bacterial growth was monitored by visual inspection, with resazurin used as a colorimetric indicator of cell viability.^32,33^ The MIC was defined as the lowest concentration of LQFM326 (**6**) that completely inhibited visible mycobacterial growth. Negative and positive controls were included on each plate. All MIC assays in this study were performed with positive controls (antibiotics), including CIP and CLA for *M. abscessus* subsp. abscessus and *M. abscessus* subsp. *bolletii*; and CLA to *M. abscessus* subsp. *massiliense*. CLA, EMB, and RMP for *M. tuberculosis* strains (H37Ra ATCC 25177) and *M. intracellulare*.

### 5.6 Analysis of the Modulatory Factor in Mycobacteria

To evaluate the potential influence of known efflux mechanisms present in mycobacteria and antimicrobial activity in *M. abscessus* subsp. *abscessus*, *M. abscessus* subsp. *bolletii*, and *M. abscessus* subsp. *intracellulare* strains, the MF assay was performed. This assay evaluates alterations in the MIC of an antimicrobial against a specific strain in the presence of EI. Initially, the reduction in the MIC of LQFM326 (**6**) (used as the antimicrobial) was assessed when combined with the classic efflux inhibitors VP or TZ.

Additionally, the compounds were assessed for potential synergism/EI, in combination with antimicrobials commonly used to treat diseases caused by these mycobacteria. The following antimicrobials were used: CIP and CLA for *M. abscessus* subsp. abscessus and *M. abscessus* subsp. *bolletii*; and CLA, EMB, and RMP for *M. intracellulare*. To calculate the MF, the following formula was applied: MF = Antimicrobial MIC/(Antimicrobial MIC + EI). An MF value ≥4, indicating a reduction of four times or greater, was considered significant.^19,20^

### 5.7 Preparation of NTM for Scanning Electron Microscopy

Colonies of *M. abscessus* subsp. *massiliense* GO06, cultivated on nutrient agar, were exposed to LQFM326 (**6**) and analyzed using Scanning Electron Microscopy (SEM). LQFM326 (**6**) was diluted in broth to its MIC concentration (31.25 μg/mL) and incubated with the mycobacteria for 24 h.^34^ After the incubation period, LQFM326 (**6**) was removed, and the cells were incubated with Karnovsky’s fixative solution (prepared with 2% paraformaldehyde, 2% glutaraldehyde in 0.01 M sodium cacodylate buffer) for 30 min at 4 °C. The fixative solution was then removed, and a series of dehydration steps were performed, followed by ethanol washes (30, 50, 70, 90, and 100%) for 10 min each. This was followed by treatments with acetone and hexamethyldisilazane (HMDS) for an additional 5 min. The colonies were then coated with a thin gold layer using a Denton Vacuum Desk V metallizer. Images were obtained using a field emission scanning electron microscope (FEG-SEM), JEOL JSM-7100F (JEOL, Japan), at an electron acceleration voltage of 3 kV in Secondary Electron Detection (SED) mode.

### 5.8 Cellular Viability

#### 5.8.1 Assess Solution Preparation

For the cytotoxicity assay, the murine fibroblast cell line L929 were cultured under standard conditions in a 37 °C incubator with 5% CO_2_ in a humidified atmosphere. To prepare for the test solution, 1 mg of LQFM326 (**6**) was dissolved in 480 μL of DMSO, followed by the addition of 23.52 μL of supplemented RPMI medium to create the stock solution. The final test solution contained a maximum concentration of 3% DMSO and 0.042 mg/mL of LQFM326 (**6**). From this stock solution, five different concentrations were assessed.

#### 5.8.2 Cell Culture

The murine fibroblast cell line L929, purchased from the Rio de Janeiro Cell Bank (BCRJ) (Rio de Janeiro, RJ, Brazil), was used for the experiments. Cells were cultured in Gibco Roswell Park Memorial Institute 1640 (RPMI) medium, supplemented with 5% Fetal Bovine Serum (FBS) and 0.01% Penicillin/Streptomycin. The cells were maintained in 75 cm^2^ culture flasks in an incubator at 37 °C, with 5% CO_2_ and 95% air, under controlled humidity. For experimentation, the cells were washed with PBS buffer, detached from the culture flasks using an EDTA solution (0.25/0.03%), and centrifuged at 1000 rpm for 5 min. The supernatant was discarded, and the pellet was resuspended in fresh culture medium for subsequent cell counting.

#### 5.8.3 “Live and Dead” Assay

L929 cells were seeded into 24-well plates at a density of 10^5^ cells/1000 μL/well and cultured for 24 h to allow cell adhesion in supplemented culture medium. After 24 h, the culture medium was removed, and the test solution was added to each well. In the first four wells, 1 mL of the test solution was added, and for every subsequent set of four wells, the amount of test solution was halved and completed with nonsupplemented RPMI medium. After adding the test solution to the wells, including the positive control, the cells were incubated at standard conditions for 24 h. Following incubation, the cells were examined under an inverted microscope and washed with sterile PBS. The cells were then incubated for 20 min with 1000 μL of PBS and 6 μL of neutral red dye. After incubation, PBS was removed, and the cells were washed again. Subsequently, 3.5 μL of Evans Blue dye was added, and the cells were incubated for another 20 min. After this period, the dye solution was removed, and PBS was added again. The wells were then photographed under an inverted microscope at 200× magnification. The viable cell count was performed using the TMArker software, which was pretrained to differentiate live and dead cells.

### 5.9 Selectivity Index

The SI was calculated using the eq Supporting Information = CC_50_/MIC, with an SI value of 10 or greater considered indicative of selectivity.^23–25^

Statistical analysis and graphical representation of cellular viability data were performed using GraphPad Prism 8. First, the Shapiro-Wilk test was conducted to assess the normality of the data. Subsequently, an ordinary one-way ANOVA was used to analyze the differences between groups. The CC_50_ values were determined using nonlinear regression. Differences were considered statistically significant when *p* < 0.05.

## Supplementary Material



## Notes

Structural characterization of LQFM326 by infrared spectroscopy, ^1^H and ^13^C NMR, HSQC, HMBC, and mass spectrometry. It also presents a representative microplate assay used to determine the MIC of LQFM326 against the *M. tuberculosis* H37Ra strain (ATCC 25177) and clinical isolates of *Mycobacterium* spp. for identification purposes (PDF)
