# Supplementary material for: In Vitro Antimycobacterial Activity Evaluation of a New Lead Compound (LQFM326) against Clinical Strains of Mycobacterium sp
Source: ACS Omega. 2025 Aug 26;10(35):39875–83. doi: 10.1021/acsomega.5c04174 (PMC12423788; doi:10.1021/acsomega.5c04174)
Supplement: Supplementary file 1 [file ao5c04174_si_001.pdf]

Supporting Information

***In vitro* antimycobacterial activity evaluation of a new lead compound (LQFM326) against clinical strains of *Mycobacterium* sp.**

Tracy M. M. Martins[a], Luciano M. Lião[b], Gerlon A. R. Oliveira[c], Pedro E. A. Silva[d], Ana J. Reis[d], Yasmin C. Neves[d], Glaura R. C. C. Lima[e], Beatriz S. Gontijo[f], José R. do Carmo Neto[f], Jonathas X. Pereira[f], André Kipnis[g], Ricardo Menegatti[h*]

[a]*Center for Morphological Studies (NEM-ATM), Faculty of Medicine (FAMED/CALTA), Federal University of Pará, Altamira, PA, Brazil*

[b]*Nuclear Magnetic Resonance Laboratory (LabRMN), Institute of Chemistry, Federal University of Goiás, Goiânia, GO, Brazil*

[c]*Department of Pharmacy, Faculty of Health Sciences, University of Brasília, Brasília, Brazil*

[d]*Laboratory of Mycobacteria, Faculty of Medicine, Federal University of Rio Grande (FAMED), Rio Grande, RS, Brazil*

[e]*Medical Biology of Central Public Health Laboratory of the Federal District (LACEN-DF), Brasília, DF, Brazil*

,[f]*Cellular and Molecular Pathology Laboratory, Institute of Tropical Pathology and Public Health (IPTSP), Federal University of Goiás, Goiânia, GO, Brazil*

[g]*Molecular Bacteriology Laboratory, Institute of Tropical Pathology and Public Health, Federal University of Goiás, Goiânia, GO, Brazil*

[h]*Laboratory of Medicinal Pharmaceutical Chemistry (LQFM), Faculty of Pharmacy, Federal University of Goiás, Goiânia, GO, Brazil*

E-mail: rm_rj@ufg.br # Authors with equal contributions

Supplementary material LQFM326

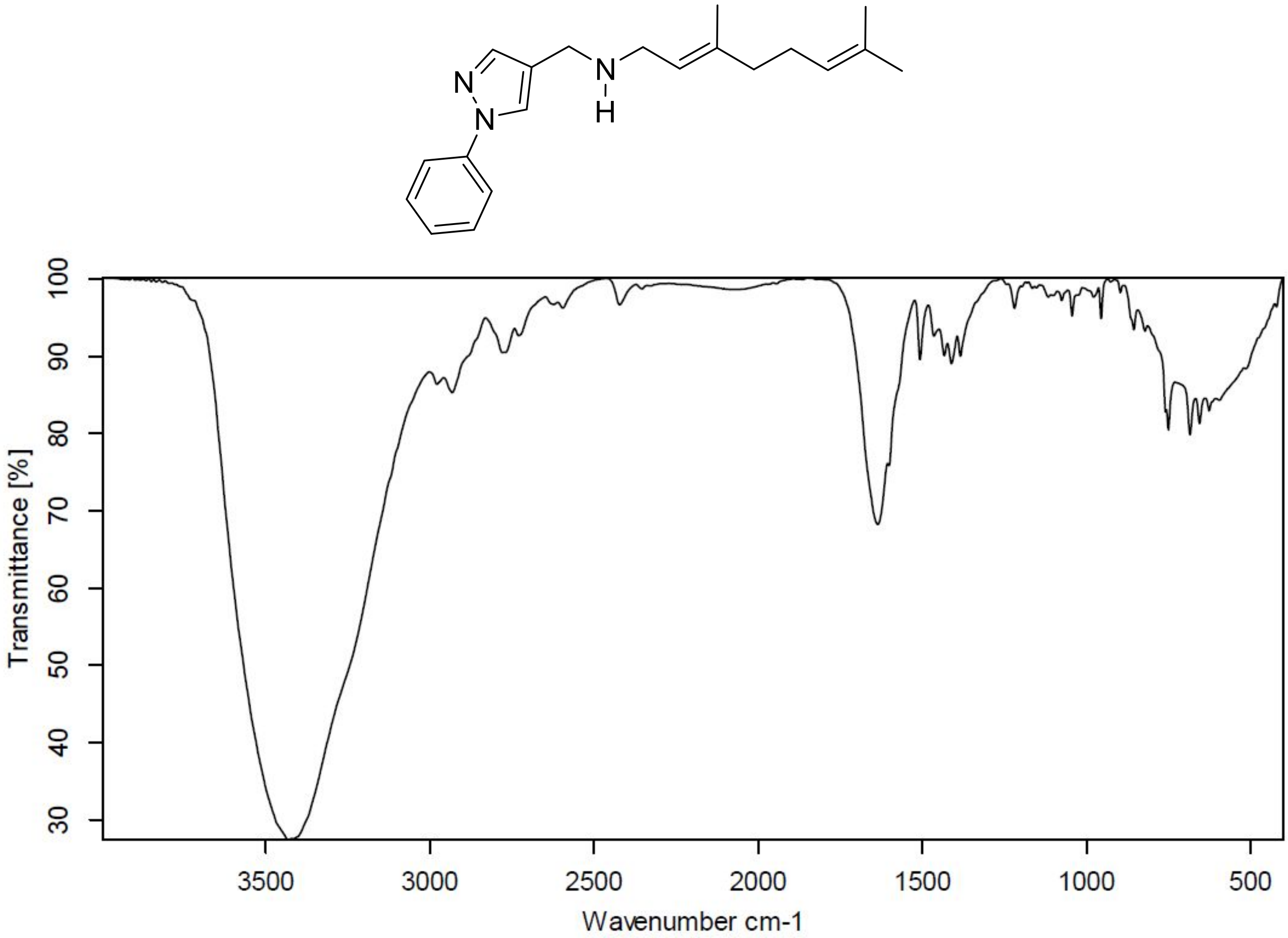

Figure S1. Infrared spectrum for (*E*)-3,7-dimethyl-N-((1-phenyl-1H-pyrazol-4-yl)methyl)octa-2,6-dien-1-amine (**6**) – LQFM326 in KBr.

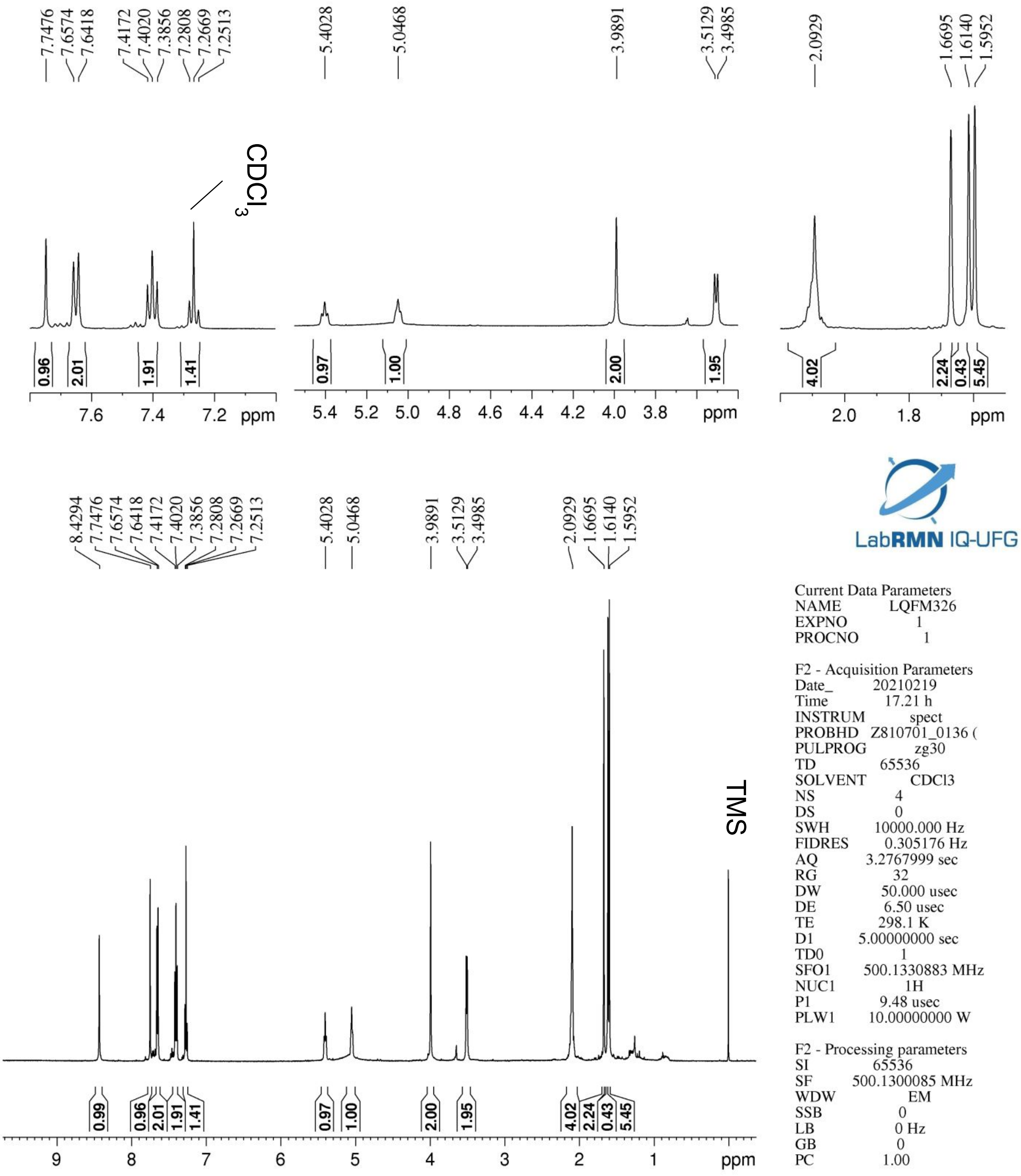

Figure S1. ^{1}H NMR spectrum for (*E*)-3,7-dimethyl-N-((1-phenyl-1H-pyrazol-4-yl)methyl)octa-2,6-dien-1-amine (**6**) – LQFM326 in $CDCl_3$.

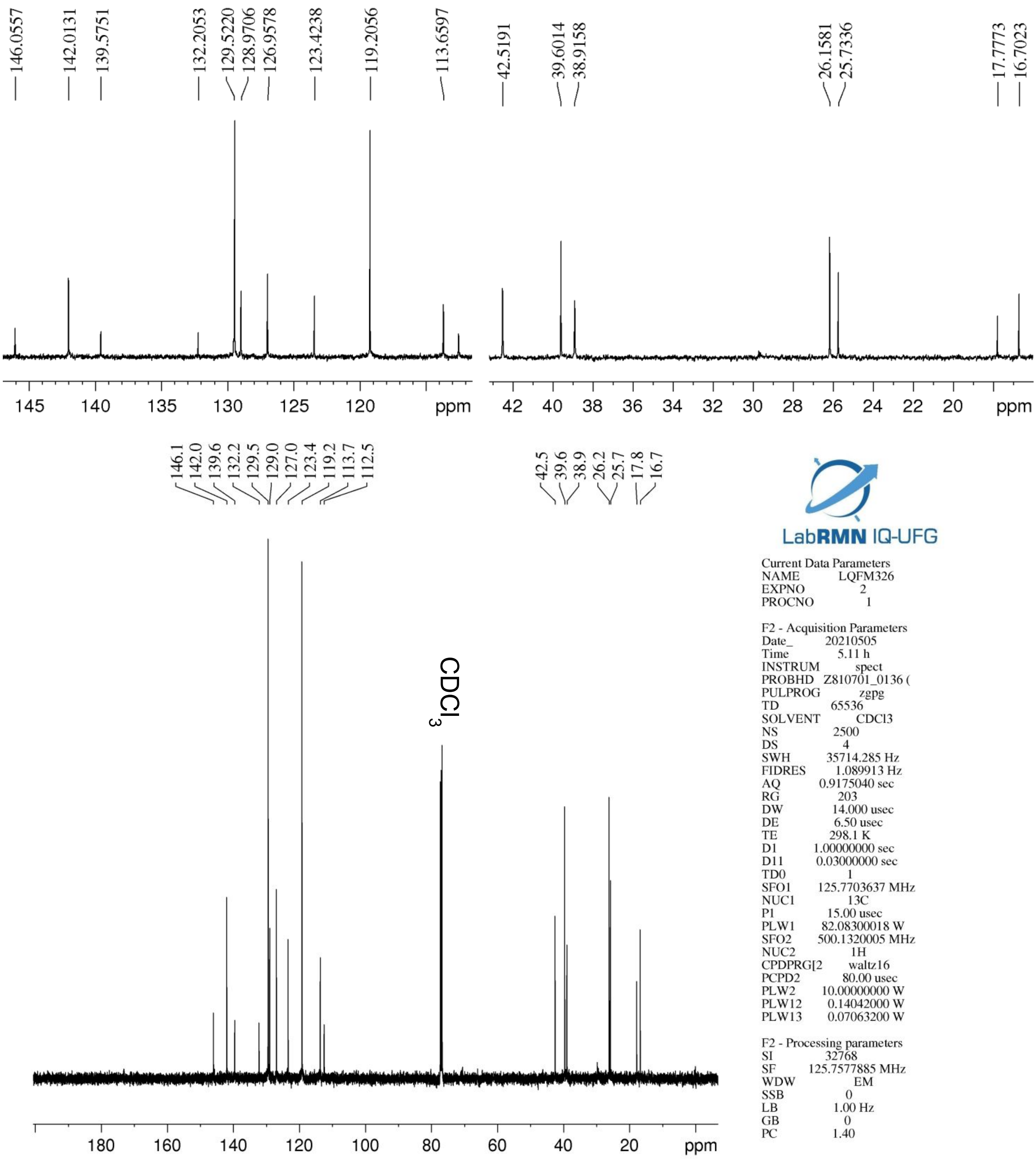

Figure S3. ^{13}C NMR spectrum for (*E*)-3,7-dimethyl-N-((1-phenyl-1H-pyrazol-4-yl)methyl)octa-2,6-dien-1-amine (**6**) – LQFM326 in $CDCl_3$.

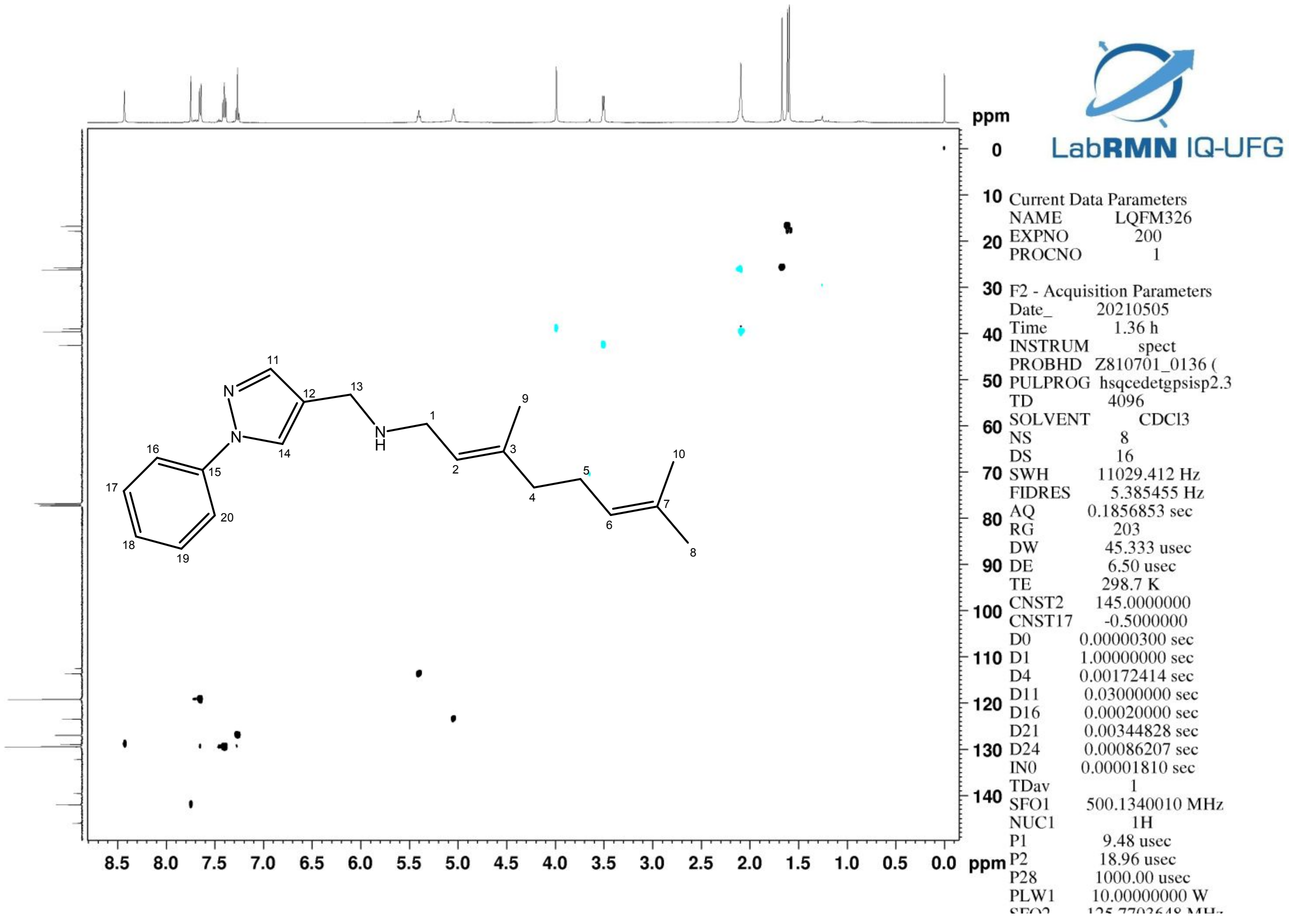

Figure S4. ^{1}H-^{13}C HSQC NMR contour map for (*E*)-3,7-dimethyl-N-((1-phenyl-1H-pyrazol-4-yl)methyl)octa-2,6-dien-1-amine (**6**) – LQFM326 in $CDCl_3$.

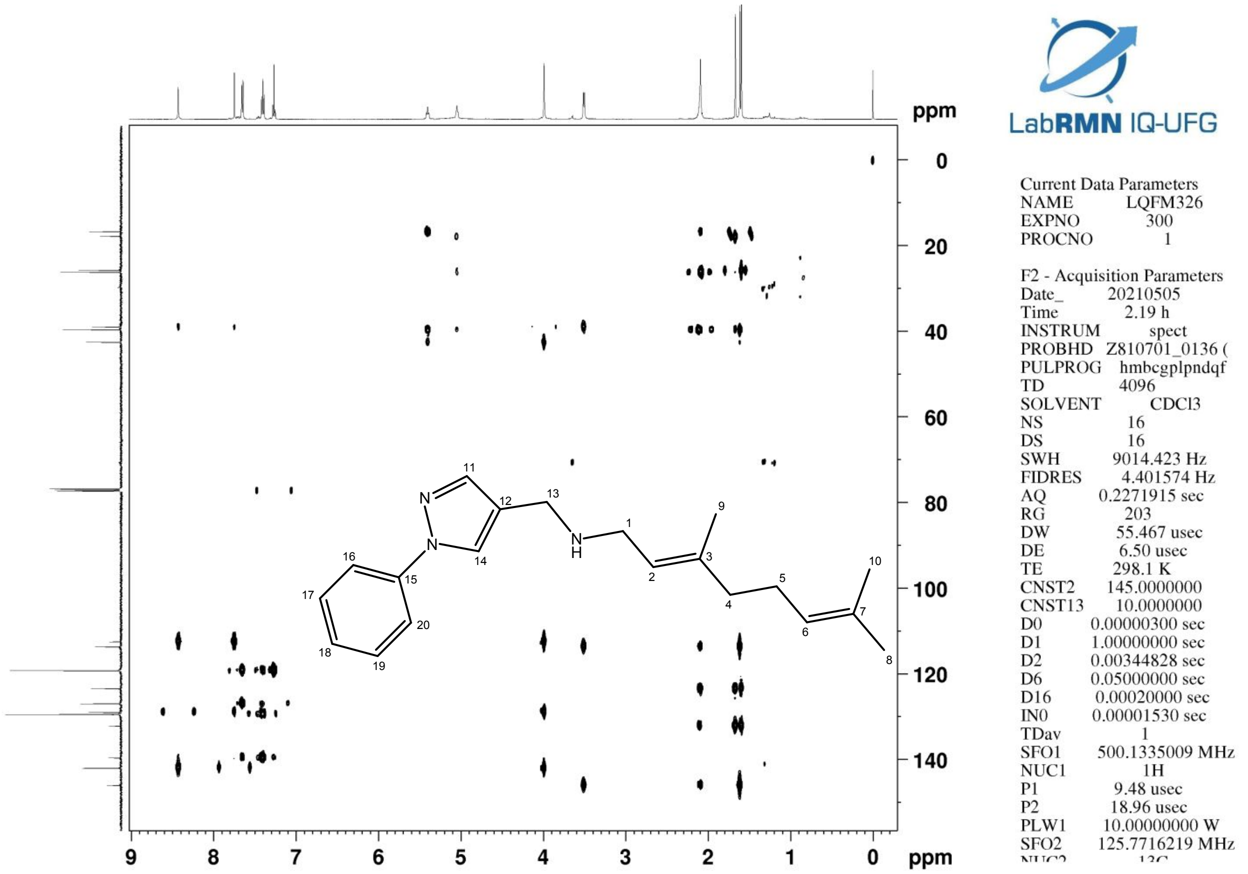

Figure S5. ^{1}H-^{13}C HMBC NMR contour map for (*E*)-3,7-dimethyl-N-((1-phenyl-1H-pyrazol-4-yl)methyl)octa-2,6-dien-1-amine (**6**) – LQFM326 in $CDCl_3$.

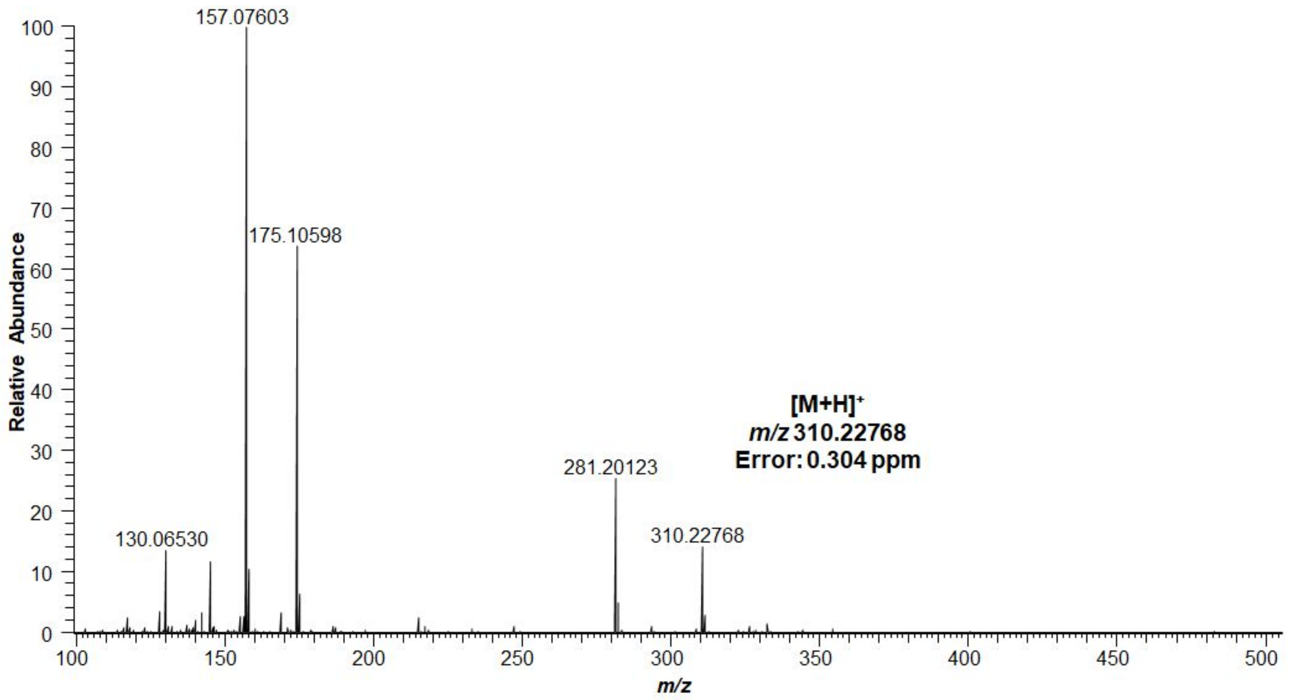

Figure S6. Mass spectrum obtained for (*E*)-3,7-dimethyl-N-((1-phenyl-1H-pyrazol-4-yl)methyl)octa-2,6-dien-1-amine (***6***) – LQFM326.

Table S1. ^{1}H and ^{13}C NMR spectral data for (*E*)-3,7-dimethyl-N-((1-phenyl-1H-pyrazol-4-yl)methyl)octa-2,6-dien-1-amine (***6***) – LQFM326 in $CDCl_3$.

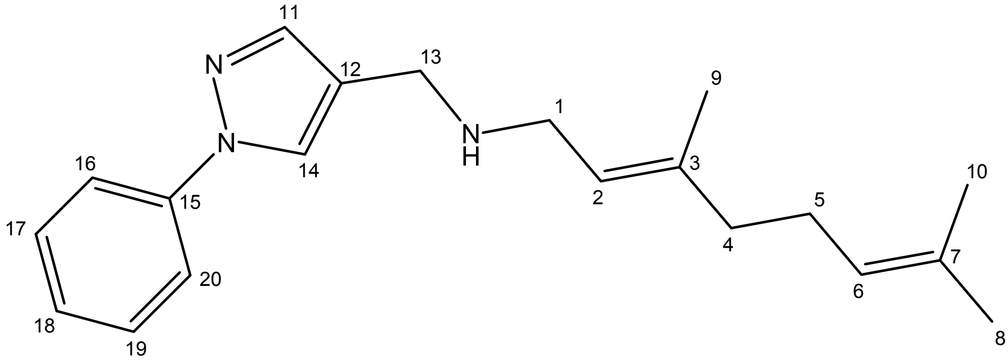

	$\delta\,^{1}H$ (*multiplicity*, *J* Hz)	$\delta\,^{13}C$
1	3.50 (*d*, 7.2)	42.5
2	5.40 (*t*, 7.2)	113.7
3	-	146.1
4	2.09 (*m*)	39.6
5	2.09 (*m*)	26.2
6	5.05 (*m*)	123.4
7	-	132.2
8	1.67 (*s*)	25.7
9	1.61 (*s*)	16.7
10	1.60 (*s*)	17.8
11	7.75 (*s*)	142.0
12	-	129.5
13	3.99 (*s*)	38.9
14	8.43 (*s*)	129.0
15	-	139.6
16; 20	7.65 (*m*)	119.2
17; 19	7.40 (*m*)	129.5
18	7.27 (*m*)	127.0

^{1}H and ^{13}C NMR assignments are based on ^{1}H and ^{1}H–^{13}C HSQC/HMBC spectra. Measured in $CDCl_3$ at 500 MHz and 125 MHz.
Multiplicities: d (doublet), m (multiplet), s (singlet), t (triplet).

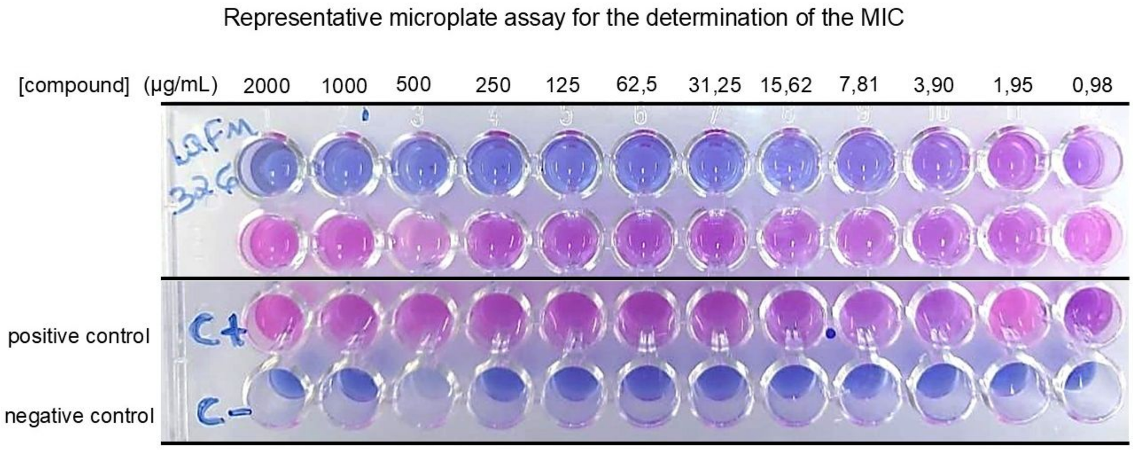

Figure S7 – Representative microplate assay for the determination of the minimum inhibitory concentration (MIC) of the compound LQFM326 (**6**), against the strain of *M. tuberculosis* H37Ra ATCC 25177. The visually determined MIC is 15.63µg/mL.

Table S2. Clinical strains of *Mycobacterium* sp. Identification.*

Species	Identification
M. abscessus subsp abscessus	*ATCC 19977*
M. tuberculosis H37Rv	*ATCC 27294*
M. tuberculosis H37Ra	*ATCC 25177*
M. abcessus subsp. *massiliense*	GO06
M. abscessus subsp. *abscessus*	*rpoB* and *hsp65* genes partial sequencing
M. intracellulare	*rpoB* and *hsp65* genes partial sequencing
M. abscessus subsp. *bolletii*	*rpoB* and *hsp65* genes partial sequencing

**Except for the ATCC strains, all strains were previously identified using the rpoB and hsp65 genes sequencing, for NTM strains, and mycobacterial interspersed repetitive unit-variable number tandem repeat 24-loci, insertion sequence (IS)6110 and whole-genome sequencing for M. tuberculosis strains*
